# Characteristics of Participants and Nonparticipants in a Blended Internet-Based Physical Activity Trial for Breast and Prostate Cancer Survivors: Cross-sectional Study

**DOI:** 10.2196/25464

**Published:** 2021-10-05

**Authors:** Hester J vd Wiel, Martijn M Stuiver, Anne M May, Susan van Grinsven, Marlou F A Benink, Neil K Aaronson, Hester S A Oldenburg, Henk G van der Poel, Wim H van Harten, Wim G Groen

**Affiliations:** 1 Division of Psychosocial Research and Epidemiology The Netherlands Cancer Institute Amsterdam Netherlands; 2 Center for Quality of Life Netherlands Cancer Institute Amsterdam Netherlands; 3 Center of Expertise Urban Vitality Faculty of Health Amsterdam University of Applied Sciences Amsterdam Netherlands; 4 Julius Center for Health Sciences and Primary Care University Medical Center Utrecht Utrecht University Amsterdam Netherlands; 5 Rijnstate Hospital Arnhem Netherlands; 6 Division of Surgical Oncology The Netherlands Cancer Institute Amsterdam Netherlands; 7 Department of Urology The Netherlands Cancer Institute Amsterdam Netherlands; 8 Rijnstate Hospital Amsterdam Netherlands; 9 Department of Health Technology and Services research University of Twente Enschede Netherlands

**Keywords:** internet-based intervention, physical activity, nonparticipants, breast cancer survivors, prostate cancer survivors, RCT

## Abstract

**Background:**

As the number of cancer survivors is increasing, it is important to be able to offer exercise and physical activity (PA)–promoting interventions that are both effective and reasonably accessible. Internet-based interventions are typically less expensive and more accessible alternatives to on-site supervised interventions. Currently, little is known about the characteristics of nonparticipants in PA promotion trials in the cancer survivorship setting, both in general and specifically in trials using internet-supported interventions.

**Objective:**

This study aims to gain insight into the characteristics associated with nonparticipation in a blended internet-based supported intervention trial to promote PA.

**Methods:**

Breast and prostate cancer survivors, 3-36 months after primary curative treatment, were invited to participate in the PABLO trial; this trial compared an internet-based intervention to enhance PA levels, with or without additional support from a physical therapist, to usual care. Participants and nonparticipants were asked to complete a comprehensive questionnaire assessing sociodemographics, fatigue, and health-related quality of life. Baseline data for participants and nonparticipants were compared using the independent Student *t* test and chi-square test.

**Results:**

The inclusion rate in the trial was 11.03% (137/1242). Of the nonparticipants, 13.95% (154/1104) completed the questionnaire. Participants were more highly educated (*P*=.04), had a paid job less often (*P*=.03), and were on sick leave more often (*P*=.03). They reported less PA per week, both moderate (*P*=.03) and vigorous (*P*<.01), before diagnosis and during leisure time (*P*<.01, effect size [ES]=0.44). They reported a significantly lower stage of change (*P*≤.01), lower self-efficacy (*P*<.01, ES=0.61), perceived barriers to PA (*P*<.01, ES=0.54), and more general fatigue (*P*<.01, ES=0.60). Participants reported lower health-related quality of life for most domains (ES ranging from 0.34 for mental health to 0.48 for social functioning). No significant differences were found for other sociodemographics, mood state, or attitudes toward or perceived social support for PA.

**Conclusions:**

The participants who self-selected for trial participation reported lower PA levels before diagnosis and a stronger need for support compared with nonparticipants. The trial thus included those patients who might benefit the most from internet-based supportive PA interventions.

**Trial Registration:**

Netherlands trial register NTR6911; https://www.trialregister.nl/trial/6733

## Introduction

### Background

Long-term side effects of cancer treatment commonly lead to a decrease in psychosocial and physical functioning [1]. Multiple systematic reviews have demonstrated the positive effects of physical exercise interventions on various outcomes in cancer patients and survivors, including fatigue, physical functioning, and health-related quality of life (HRQoL) [2-5]. There is also some evidence that exercise can have a positive effect on survival in several cancer population (eg, breast and prostate cancer) [6]. For these reasons, physical exercise programs are becoming an increasingly important component of cancer care, both during and after primary treatment [7].

As the number of cancer survivors is increasing, it is important to be able to offer exercise- and physical activity (PA)-promoting interventions that are both effective and reasonably accessible. Supervised interventions have proven to be superior to unsupervised interventions in increasing PA levels [2]. Nevertheless, previous studies have reported that approximately half of eligible patients declined to participate in supervised exercise and PA-promoting interventions [8,9]. Moreover, offering supervised exercise to all patients would represent a significant burden to the health care system in terms of financial and human resources [10]. Internet-based interventions are typically less expensive and more accessible alternatives for those who cannot or do not want to participate in on-site supervised interventions or who have limited exercise support needs. At the same time, internet-based interventions may not be suitable for every patient. An increased understanding of reasons for nonparticipation in exercise interventions, especially those that are internet-based, is required to improve selection for and referral to such programs.

Given that participation in exercise and PA promotion trials for people living with and beyond cancer could be improved, such trials also offer opportunities to study factors associated with nonparticipation. Currently, little is known about the characteristics of nonparticipants in PA promotion trials in cancer survivorship in general, and specifically in trials using internet-based interventions. Two previous studies compared the characteristics of patients with breast cancer who took part in a randomized controlled trial (RCT) of supervised exercise during radiotherapy and chemotherapy with those did not participate in the trial. Both studies reported significantly higher fatigue levels at baseline for nonparticipants [11,12]. Travel distance and time investment (eg, fixed training schedules) were also noted as reasons for nonparticipation. During chemotherapy, nonparticipants differed in attitudes toward PA; they perceived fewer benefits and more barriers and had a lower sense of self-efficacy with regard to exercising [11]. In a supervised RCT among cancer survivors [13], nonparticipants reported a lower educational level, were more likely to smoke, had higher levels of psychological distress and lower outcome expectations, and experienced fewer barriers compared with the participants.

### Objectives

To inform clinical practice and to achieve higher inclusion rates in future internet-based intervention studies, it is of interest to know more about potential patient- and tumor-specific participant and nonparticipant characteristics. It is also of interest to know whether reasons for nonparticipation in supervised programs differ from those presented with an internet-based approach in which barriers such as travel distance and strict time management are no longer relevant [14]. The aim of this study is to gain insight into the characteristics of participants and nonparticipants of an internet-based intervention promoting PA among breast and prostate cancer survivors whose primary oncological treatment had been completed between 3 months and 3 years earlier.

## Methods

### Design and Study Population

For this cross-sectional investigation, we used baseline data from the PABLO study, an RCT in which a web-based intervention is being evaluated as a means of improving PA levels in cancer survivors. Patients were recruited from 3 Dutch hospitals: the Netherlands Cancer Institute, Amsterdam, Rijnstate Hospital, Arnhem, and the University Medical Centre, Utrecht. Breast and prostate cancer survivors were randomized into 3 groups: (1) internet-based physical activity support program (IPAS), (2) IPAS + additional telephone support from a physical therapist, or (3) control group (usual care) A detailed description of the trial protocol and internet-based intervention has been published previously [15]. This protocol followed the CONSORT-EHEALTH guidelines [16].

Breast and prostate cancer survivors who had completed primary curative treatment 3-36 months earlier, but who could still be receiving adjuvant endocrine treatment or trastuzumab, were invited to participate. Patients were excluded if they lacked basic proficiency in Dutch, had serious cognitive or psychiatric problems that would preclude following the intervention, complete the study questionnaires, or lacked access to the internet. Those without a digital ID, the Dutch digital authentication system on the basis of one’s social security number (used primarily for governmental services), were also excluded, as this was required to log on to the IPAS. Patients participating in concurrent studies or rehabilitation programs containing psychosocial or exercise interventions were excluded, as were those who were unable to perform unsupervised exercise at the recommended levels or who could not safely perform such exercise according to the pre-exercise screening recommendations of the American College of Sports Medicine [17]. Patients with cardiovascular, metabolic, or renal diseases could only participate after receiving approval from their treating physician. Finally, to ensure that the trial targeted those who could potentially benefit from PA, we excluded patients who reported already engaging regularly in >200 minutes per week of moderate-to-vigorous PA for more than 6 months, as determined via a brief interview.

For this study, eligible patients who declined to participate in the PABLO trial were asked to complete a web-based questionnaire. Participants completed the same questionnaire as part of the baseline measurement. The questionnaire was administered using the web-based program *Exploratio* (Newcom Research & Consultancy). Patients who did not wish to complete the full questionnaire were offered the opportunity to voluntarily report reasons for nonparticipation on the response card that was attached to the trial invitation.

### Procedure

Patients’ medical records were screened for inclusion and exclusion criteria, except for prescreening PA levels. Potentially eligible participants for the trial were approached by mail or in person when their treating health care worker (nurse practitioner or physician [assistant]) considered the patient to be eligible for the trial. All participants and nonparticipants in this study provided written informed consent and completed the web-based questionnaire. Ethical approval was obtained from the institutional review board of the Netherlands Cancer Institute, Amsterdam (NL62269.031.17)*.*

### Outcome Measures

#### Self-reported Reasons for Nonparticipation in the PABLO Trial

Reasons for nonparticipation for those who were willing to complete the nonparticipants’ questionnaire were assessed by five preset options: (1) participation in another trial, (2) no time, (3) the study is not applicable to me or no interest, (4) participation is too burdensome for me, and (5) other.

Those who declined to complete the full nonparticipants’ questionnaire were asked if they were willing to provide the reasons for nonparticipation, using five slightly different response options: (1) I am already sufficiently physically active, (2) no time, (3) my physical state is not good enough, (4) I do not think I will benefit from it, (5) other.

#### Clinical Characteristics

Clinical data, including tumor type and staging, type of treatment, and time between diagnosis and the end of treatment, were obtained from the medical records.

#### Sociodemographics and Health Behavior

Sociodemographic information about age, sex, educational level, living and work situation, as well as lifestyle data, such as smoking behavior, alcohol consumption, and PA behavior, before the diagnosis of cancer was assessed via a questionnaire. The questionnaire also included study-specific questions about patients' use of the internet and their level of computer skills.

#### Self-reported PA, Fatigue, Mood, and Health-Related Quality of Life

Self-reported PA behavior was assessed using the International Physical Activity Questionnaire (IPAQ). The IPAQ contains 4 domains: PA at work, during transport, at home, and during leisure time. Scores were calculated according to the IPAQ manual, resulting in metabolic equivalent of task minutes per week, as the total score per domain [18].

Fatigue was assessed using the Multidimensional Fatigue Inventory Questionnaire (MFI) [19]. The MFI consisted of 20 items organized into five dimensions: general fatigue, physical fatigue, mental fatigue, reduced activity, and reduced motivation. Scores ranged from 4 to 20 per subscale. Higher scores indicate higher levels of fatigue.

Mood was assessed using the Profile of Mood States (POMS) [20]. This 32-item questionnaire consisted of five mood scales: anger, depression, fatigue, tension, and vitality. For anger, depression, fatigue, and tension, higher scores indicate higher mood expression of a specific item (ranging from 0 to 20). Vitality was reverse coded so that higher scores indicated less vitality (ranging from 0 to 20). Items’ scores ranged from 0 to 4. The total score was calculated as the sum of the means of the 4 mood scales minus the vitality score. Higher scores indicate higher levels of anger, tension, depression, fatigue, and lower vitality.

HRQoL was assessed using the 36-Item Short Form Health Survey (SF-36) [21]. The SF-36 includes eight scales assessing physical functioning, vitality, role functioning limitations due to physical problems, role functioning limitations due to emotional problems, social functioning, physical pain, mental health, and general health. Scores range from 0 to 100 per subscale. Higher scores indicated higher levels of functioning and HRQoL.

#### Behavioral and Attitudinal Variables Toward PA

The current exercise behavior stage was assessed by a single item, on the basis of the transtheoretical model [22]. Patients were asked to choose from five statements, each of which corresponded to one of the stages of change, the one statement that best described their current situation. In the transtheoretical model, five behavioral change stages are identified: (1) precontemplation (ie, not sufficiently active and not intending to change; (2) contemplation (ie, not sufficiently active but willing to change within the next 6 months); (3) preparation (ie, not sufficiently active but planning to change within 1 month; (4) action (ie, sufficiently active but for <6 months); and (5) maintenance (ie, sufficiently active for >6 months) [22].

Questions on the basis of the theory of planned behavior were used to assess self-efficacy, barriers to and benefits of PA, and perceived social support [11,23]. Five items assessed self-efficacy regarding PA. Respondents rated on a 0-10 response scale, how likely they thought it was that they would exercise when tired, in a bad mood, when feeling pressed for time, when on holiday, or with bad weather [24]. The overall self-efficacy score was obtained by calculating the average of all items, ranging from 0 to 10. A higher score indicates a stronger sense of self-efficacy. Cronbach α for this scale in our sample was .85.

Items on perceived barriers to and benefits of PA were selected from 2 existing questionnaires [23,24], as previously used by Van Waart et al [11]. Potential barriers were assessed using 18 items assessing motivation, money, time, energy, other obligations, transportation, support for exercise, counseling about exercise, limited possibilities in the environment, pleasure, family obligations, fear of injuries, discipline, health conditions, nausea, fatigue, pain, and work responsibilities. Responses were on a 5-point Likert-type scale (*never a barrier* to *very often a barrier*). The barrier score was calculated as the average of the item scores, ranging from 0 to 5 per item. Higher scores indicate a higher perceived level of barriers. Cronbach α for the total scale was .87.

The perceived benefits of PA were assessed using 11 items, including improved health leading to a reduced risk of disease, feeling better about oneself, improved fitness, improved daily functioning, weight loss, meeting new people, getting one’s mind off cancer and its treatment, improving overall well-being, coping with the stress of cancer and treatment, gaining control over cancer and life, and recovering from treatment. Items were scored on a 5-point Likert scale (*completely disagree* to *completely agree*). The perceived benefit score was obtained by averaging item scores, ranging from 0 to 5 per item. A higher score indicated a higher sense of benefit. Cronbach α for this scale was .91.

Attitudes toward PA were assessed using 7-point adjective rating scales. Two dimensions were measured: (1) instrumental attitude (useful–useless, harmful–beneficial, wise–foolish, and bad–good) and (2) affective attitude (enjoyable–unenjoyable, boring–interesting, pleasant–unpleasant, and easy–hard) [23]. The overall score for attitude was similarly calculated as the average score of the combined 8 items, ranging from 0 to 7 per item. Cronbach α for this scale was .95. Higher scores indicate more positive attitudes toward exercise [11].

Finally, perceived social support from partners, family, friends, colleagues, general practitioners, treating physicians, and other patients with cancer for PA was assessed. These items were scored on a 5-point Likert-type response scale, with an overall Cronbach α of .9. The overall perceived support score was calculated by summing the items [11,25]. The higher the score, the more perceived social support.

### Statistical Analysis

We report descriptive statistics using means, SDs, medians, and IQRs for continuous variables, and frequencies and percentages for categorical variables.

We compared baseline data between participants and nonparticipants using an independent Student *t* test for continuous variables. For ordinal variables, a linear-by-linear association was used. For dichotomous variables, we used Fisher exact test.

On the basis of the literature, we hypothesized that there might be an interaction between tumor type and the following variables—age, work situation, PA levels before diagnosis, IPAQ-scores, and stage of change. Interaction tests were performed using regression analysis. In case of a statistically significant interaction, the descriptive statistics and group comparisons were stratified by tumor type. Two-sided *P* values <.05 were considered statistically significant. Effect sizes were calculated as the group mean differences divided by pooled SD. Given the exploratory nature of this study, we did not correct for multiple tests. All analyses were performed using SPSS version 25 (SPSS Inc).

## Results

### Participation of Respondents

**Figure 1:**
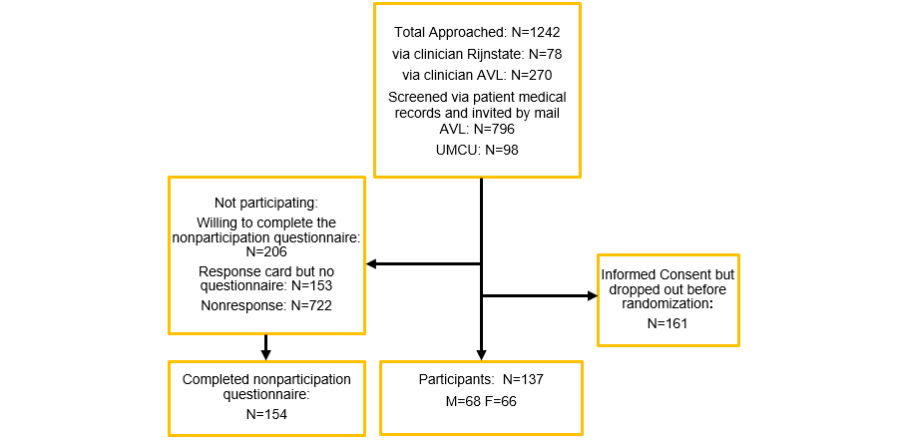
Diagram of nonparticipants of the PABLO trial. AVL: Antoni van Leeuwenhoek; F: Female; M: Male; UMCU: University Medical Centre Utrecht.

Of the 1242 invited individuals, 137 participated in the PABLO trial (participation rate: 137/1242, 11.03%). Of all nonparticipants (n=1105), 206 indicated a willingness to complete the questionnaire, of whom 154 actually did so (154/1105, 13.94% response rate). More than half of the patients (722/1242, 58.13%) did not respond. Another 12.32% (153/1242) of the invited patients sent back a response card, including reasons for not participating in the trial, but did not complete the web-based questionnaire (Figure 1).

### Self-reported Reasons of Nonparticipation

The most often reported reason for nonparticipation was the perceived adequate level of PA. This was reported by 40.3% (62/154) of those who completed the questionnaire and 82.5% (127/154) of those who provided their reason on the response card. Additional reasons reported by the questionnaire respondents were (multiple options possible): “I don’t have time to participate” (33/154, 21.4%), “Participation is too burdensome for me” (6/154, 3.9%), “The study is not applicable to me/no interest” (4/154, 2.6%), “Participation in another trial” (3/154, 1.9%) and “Other” (46/154, 29.9%). Other reasons stated on the response card by those who did not complete the questionnaire were “I don’t think I will benefit from it” (16/198, 8.1%), “No time” (15/198, 7.6%) “My physical state is not good enough” (8/198, 4%) and “Other” (41/198, 20.7%), of which 3 reported “The online approach.”

### Clinical Characteristics

Statistically significant interactions with tumor type were observed for age, retirement, and self-rating of a vigorous level of PA on the IPAQ. For these variables, stratified results were reported, in addition to the total group results.

The percentage of nonparticipants did not differ significantly between breast and prostate cancer survivors (82/154, 53.3% and 72/154, 46.7%, respectively). In breast cancer survivors, nonparticipants were less likely to have undergone a mastectomy and were more likely to have undergone breast-conserving surgery. No significant differences in any treatment-related variables were observed within the prostate cancer survivor group (Table 1).

**Table 1:** Baseline clinical characteristics of 154 individuals who filled out the nonparticipants questionnaire and 137 participants.

Clinical characteristic			Nonparticipants breast cancer (n=82)		Participants breast cancer (n=67)		Nonparticipants vs participants breast cancer, *P* value		Nonparticipants prostate cancer (n=72)		Participants prostate cancer (n=70)		Nonparticipants vs participants prostate cancer, *P* value
Tumor type or sex, n (%)			82 (53.2)		67 (51.1)		N/A^a^		72 (46.8)		70 (48.9)		N/A
**Treatment, n (%)^b^**													
	Chemotherapy	32 (39.0)		28 (44.4)		.52		0 (0)		0 (0)		N/A	
	Radiotherapy	64 (78.0)		48 (71.6)		.46		24 (33.3)		20 (28.6)		.58	
	Chemo and radiotherapy	26 (31.7)		22 (34.9)		.69		0 (0)		0 (0)		N/A	
	Endocrine therapy	51 (62.2)		32 (47.8)		.44		6 (8.3)		6 (8.8)		.92	
	Breast-conserving surgery	65 (79.3)		41 (61.2)		.02		N/A		N/A		N/A	
	Mastectomy	16 (19.5)		24 (35.8)		.03		N/A		N/A		N/A	
	Breast reconstruction	24 (29.3)		24 (35.8)		.40		N/A		N/A		N/A	
	Prostatectomy	N/A		N/A		N/A		48 (66.7)		52 (75.4)		.26	
	Brachytherapy	N/A		N/A		N/A		3 (4.2)		2 (3.0)		.71	
Treatment duration (months), mean (SD)			6.8 (4.8)		6.7 (4.1)		.92		4.1 (3.6)		4.5 (5.3)		.61

^a^N/A: not applicable.

^b^Combination of treatments possible per patient, total percentages reach above 100%.

### Sociodemographics and Health Behavior at Baseline

The mean age of the participants was 60.1 years (SD 14.1). The mean age of the nonparticipants was 63 years (SD 11.1). In breast cancer survivors, nonparticipants were significantly older than participants (mean 57.35 vs 52.66%; *P*=.01). For the total group, nonparticipants had significantly lower education levels than did the participants (*P*=.04). No significant differences between nonparticipants and participants were found in living situations. Nonparticipants more often had paid jobs (*P*=.03) and were less on sick leave (*P*=.03). Nonparticipating prostate cancer survivors were more often retired (*P*=.03) than the participants. No significant differences were found between the groups in terms of smoking or alcohol consumption. Self-reported computer skills and frequency of internet use did not differ significantly between groups. Nonparticipants more often reported being moderately (*P*<.001) and vigorously (*P*<.001) physically active per week in the period before diagnosis than the participants (Table 2).

**Table 2:** Sociodemographics at baseline of 154 individuals who filled out the nonparticipants questionnaire and 137 participants.

Sociodemographic			Nonparticipants (n=154)		Participants (n=137)		Nonparticipants vs participants, *P* value	
Age (years), mean (SD)			63 (11.1)		60.1 (14.1)		.05	
**Living situation, n (%)**								.53
	Single	19 (12.3)		23 (16.8)				
	Living together	128 (83.1)		108 (78.8)				
	With partner, not living together	6 (3.9)		5 (3.6)				
	Missing	1 (0.6)		1 (0.7)				
**Education level (%)**								.04
	Primary school	2 (1.3)		2 (1.5)				
	High School	68 (44.1)		46 (33.6)				
	College or university	80 (52.0)		88 (64.2)				
	Missing	4 (2.6%		1 (0.7)				
**Work situation, n (%)^a^**								
	Paid job	76 (49.4)		56 (42.4)		.03		
	Retired	67 (45.6)		45 (33.1)		.03		
	Sick leave	8 (5.2)		16 (11.7)		.03		
	Other^b^	42 (27.3)		40 (30.1)		.95		
**Smoking behavior, n (%)**								.10
	Never	56 (36.4)		62 (45.3)				
	Quit	82 (53.2)		64 (45.9)				
	Current	16 (10.4)		10 (6.8)				
	Missing	N/A^c^		1 (0.7)				
**Alcohol consumption, n (%)**								.09
	No	27 (17.5)		35 (27.1)				
	Yes	127 (82.5)		101 (72.9)				
	Missing	N/A		1 (0.7)				
**Computer use, n (%)**								.81
	Sometimes	7 (4.5)		7 (5.1)				
	Often	146 (94.8)		128 (93.4)				
	Missing	1 (0.6)		2 (1.5)				
**Computer skills, n (%)**								.66
	Bad	14 (9.1)		11 (8.0)				
	Moderate	43 (27.9)		36 (26.3)				
	Good	96 (62.3)		88 (64.2)				
	Missing	1 (0.6)		2 (1.5)				
**Physical activity levels before diagnosis^d^ (in days per week), mean (SD)**								
	Moderate^e^	6.4 (1.9)		5.7 (2.4)		<.001		
	Vigorous^f^	4.0 (2.2)		2.9 (2.1)		<.001		

^q^Multi-answer options, total percentage reaches above 100%; Missings: paid job, Nonparticipants n=17, participants n=5; Retired, nonparticipants n=7, participants n=1; At home because of illness: nonparticipants n=13, participants n=5; Other, nonparticipants n=16, participants n=1.

^b^Student, voluntarily unemployed, involuntarily employed, volunteer work.

^c^N/A: not applicable.

^d^Effect size for physical activity levels before diagnosis: moderate, 0.32; vigorous, 0.51.

^e^Question: How many days of the week were you moderate physical active for at least 30 minutes?

^f^Question: How many days of the week were you vigorous physical active for at least 20 minutes?

### Self-reported PA, Fatigue, Mood, and Health-Related Quality of Life

As shown in Table 3, we did not observe a significant difference between participants and nonparticipants for PA intensities, as measured by the IPAQ (ie, walking, moderate, or vigorous). For the IPAQ domain leisure time, participants were significantly less active (metabolic equivalent of task minutes per week) than nonparticipants (*P*<.01, ES=0.44). No significant differences were found for the other 3 IPAQ domains (ie, at work, home, and during transport). In the stratified analysis (data not shown in the table), we observed significantly lower levels of vigorous PA in participating breast cancer survivors (*P*=.01, ES=0.45). This difference was not observed in prostate cancer survivors. No significant differences were observed between participants and nonparticipants in any of the five domains of mood states. Trial participants reported significantly more fatigue than nonparticipants on all five dimensions of the MFI: general fatigue (*P*<.01, ES=0.60), physical fatigue (*P*<.01, ES=0.77), reduced activity (*P*<.01, ES=0.61), mental fatigue (*P*<.01, ES=0.45), and reduced motivation (*P*=.02, ES=0.30). For HRQoL, participants reported significantly worse scores for nearly all domains of the SF-36, with effect sizes ranging from 0.34 for mental health to 0.48 for social functioning. Emotional role functioning was the only domain in which no significant group differences were found (Table 3).

**Table 3:** Group differences in descriptive statistics for the outcome measures of fatigue, quality of life, mood status, PA levels, and PA attitude of 154 individuals who filled out the nonparticipants questionnaire and 137 participants.

Measure			Nonparticipants (n=154)		Participants (n=137)		Mean difference (95% CI)		Effect size		Nonparticipants vs participants, *P* value	
**IPAQ^a^—intensity, mean (SD)**												
	Walking	1630.9 (1758.9)		1278.9 (1800.1)		–343.0 (–755.7 to 69.6)		0.20		.10		
	Moderate physical activity	4190.4 (3898.5)		3522.25 (4225.4)		–668.1 (1609.0 to 272.7)		0.15		.16		
	Vigorous physical activity	1699.7 (3063.1)		1091.8 (2882.9)		–607.9 (–1299.6 to 83.7)		0.21		.09		
**IPAQ^a^—per domain, mean (SD)**												
	Work	1443.6 (3890.1)		1611.9 (4454.1)		168.3 (–797.9 to 1134.4)		0.05		.68		
	At home	2427.0 (2752.4)		1984.6 (3200.5)		–442.4 (–1131.7 to 246.8)		0.15		.20		
	Leisure time	2260.8 (2686.5)		1148.4 (2300.5)		–1112.3 (–1646.8 to 577.9)		0.44		<.01		
	During transport	1430.2 (1505.0)		1187.1 (1493.0)		–243.2 (–591.0 to 104.6)		0.16		.17		
**MFI^b^, mean (SD)**												
	General fatigue	9.7 (4.2)		12.3 (4.5)		2.7 (1.7 to 3.7)		0.60		<.01		
	Physical fatigue	8.8 (4.3)		12.1 (4.3)		3.4 (2.4 to 4.4)		0.77		<.01		
	Reduced activity	9.0 (3.9)		11.3 (4.0)		2.3 (1.4 to 3.3)		0.61		<.01		
	Mental fatigue	8.3 (3.6)		10.0 (4.0)		1.7 (0.8 to 2.5)		0.45		<.01		
	Reduced motivation	8.8 (3.4)		9.8 (3.2)		1.0 (0.2 to 1.7)		0.30		.02		
**POMS^c^, mean (SD)**												
	Fatigue	0.6 (1.6)		1.0 (2.3)		0.4 (0.2 to –0.04)		0.20		.08		
	Tension	0.5 (1.5)		0.7 (1.7)		0.3 (–0.1 to 0.6)		0.13		.19		
	Depression	0.6 (2.1)		0.6 (1.8)		–0.01 (–0.5 to 0.4)0.0		0.00		.96		
	Anger	0.5 (1.8)		0.6 (1.7)		(–0.4 to 0.4)		0.06		.99		
	Vitality	15.9 (2.9)		16.5 (3.0)		0.4 (–0.3 to 0.9)		0.09		.07		
	Total	18.2 (7.5)		19.4 (7.2)		1.24 (–0.47 to 2.9)		0.16		.16		
**SF-36^d^, mean (SD)**												
	Physical functioning	88.5 (15.9)		82.6 (16.1)		–6.0 (–9.7 to –2.2)		0.37		<.01		
	Social functioning	87.0 (17.5)		77.3 (22.7)		–9.7 (–144 to –5.1)		0.48		<.01		
	Physical role	77.4 (36.2)		58.9 (43.0)		–18.5 (–27.7 to –9.4)		0.47		<.01		
	Vitality	70.7 (19.0)		59.7 (20.4)		–11.0 (–15.61 to –6.5)		0.56		<.01		
	Emotional role	84.4 (32.2)		78.5 (33.7)		–5.9 (–13.5 to 1.7)		0.19		.13		
	Mental health	80.7 (15.3)		74.8 (19.1)		–5.9 (–9.8 to -1.9)		0.34		<.01		
	General health	67.9 (17.7)		60.9 (20.8)		–7.0 (–11.5 to –2.6)		0.36		<.01		
	Bodily pain	85.9 (16.7)		78.9 (20.4)		–7.0 (–11.5 to –2.6)		0.38		<.01		
**Stage of change, n (%)**												<.01
	Precontemplation	1 (0.6)		2 (1.5)		N/A^e^		N/A		N/A		
	Contemplation	5 (3.2)		19 (13.9)		N/A		N/A		N/A		
	Preparation	20 (13.0)		43 (31.4)		N/A		N/A		N/A		
	Action	10 (6.5)		20 (14.6)		N/A		N/A		N/A		
	Maintenance	115 (74.7)		51 (37.2)		N/A		N/A		N/A		
	N/A	0 (0)		2 (1.5)		N/A		N/A		N/A		
Self-efficacy, mean (SD)			8.0 (1.8)		6.8 (2.1)		–1.1 (–1.6 to –0.7)		0.61		<.01	
Barriers, mean (SD)			1.7 (0.5)		2.0 (0.6)		0.3 (0.2 to 0.4)		0.54		<.01	
Benefits, mean (SD)			3.9 (0.8)		3.8 (0.7)		–0.1 (–0.2 to 0.1)		0.13		.46	
Attitude, mean (SD)			5.8 (1.1)		5.6 (1.0)		–0.2 (–0.4 to 0.1)		0.19		.15	
Social support, mean (SD)			4.7 (0.9)		4.6 (0.9)		–0.2 (–0.4 to 0.1)		0.11		.14	

^a^IPAQ: International Physical Activity Questionnaire scores represent total metabolic equivalent of task minutes per week.

^b^MFI: Multidimensional Fatigue Inventory Questionnaire scores range from 4 to 20, high scores indicate high fatigue.

^c^POMS: Profile of Mood States scores; see *Methods*.

^d^SF-36: 36-Item Short Form scores range 0-100, high scores indicate a better experienced quality of life.

^e^N/A: not applicable.

### PA-Related Behavioral and Attitudinal Variables

Participants reported a significantly lower stage of change (*P*<.01), lower level of self-efficacy (*P*<.01, ES=0.61), and more perceived barriers to starting with or continuing PA (*P*<.01, ES=0.54) than trial nonparticipants. We did not observe any significant group differences in attitudes toward PA or perceived social support for PA (Table 3).

## Discussion

### Principal Findings

In this study, we examined in detail the differences in characteristics between participants and nonparticipants in an internet-based PA promotion trial for breast and prostate cancer survivors. The results suggest that trial participants were a self-selected group of survivors who experienced a stronger need for support to become more physically active. Trial participants generally reported significantly lower levels of PA behavior before diagnoses and were more often in the lower stage of the behavioral stage of PA change. At the same time, they reported a higher level of symptom burden, lower HRQoL, lower self-efficacy, and more barriers to PA than nonparticipants.

Our findings are in contrast with the results of earlier studies of nonparticipants in (supervised) exercise trials during and shortly after cancer treatment, which indicated that patients with more perceived barriers to PA were more prone to decline participation [8,11,13,26]. This discrepancy could indicate that symptoms such as fatigue and experienced barriers to becoming or staying physically active during and shortly after treatment may initially contribute to lower participation rates but may result in a greater willingness to participate when a trial is introduced longer after the oncological treatment has been completed. Self-selection for participation appears to result in a study population of cancer survivors with relatively higher levels of symptom burden, lower PA levels, and more barriers to PA. Factors that might explain this self-selection within our group of survivors could be (1) the unsupervised and internet-based nature of the intervention, (2) the timing of the intervention, (3) the method of providing information during recruitment, and (4) a more general awareness of the benefits of PA. In the following paragraphs, we discuss each of these issues separately.

### The Internet-Based and Unsupervised Nature of the Trial Intervention

Use of (blended) internet-based interventions without the need for formal, hands-on supervision may have had a positive impact on trial participation by increasing the accessibility and convenience of the intervention. This might be particularly important for survivors with higher symptom burden, lower HRQoL, and more practical barriers to participation (eg, travel distance and fixed time schedules that characterize supervised exercise programs) [11,13]. Conversely, the web-based nature of the intervention was mentioned only three times as a reason for not participating in the trial. Importantly, we did not observe any significant differences in self-reported computer skills or frequency of weekly internet use between participants and nonparticipants.

### Timing of the Intervention

Eligible patients were invited to participate in the trial 3-36 months after completion of their primary treatment. In trials during treatment, patients who experienced direct side effects and distress because of treatment planning may have declined to participate in an exercise trial, those who have completed their treatment may feel that the timing is appropriate for participating in an exercise trial. In contrast, it is conceivable that because, for a substantial number of survivors, the program was offered relatively late in their survivorship trajectory, many no longer perceived a need for a PA intervention. This could indicate that many survivors are able to regain satisfactory levels of PA without the support of a formal program.

### Type of Trial Information

To obtain sufficient contrast, the trial specifically focused on survivors with insufficient self-reported levels of PA at the start of the trial. Therefore, we provided extensive information about the intervention to the target group during the recruitment process. This strategy of information provision during accrual could have generated a self-selection of survivors with relatively low levels of PA.

### General Awareness of the Benefits of PA

Information available about the potential benefits of PA has increased over the last few years. Such information is available both as part of routine hospital care and through public communications about specified exercise guidelines for cancer survivors [27]. This may have led to more awareness among cancer survivors about the importance of being physically active. As a result, some survivors may have increased their levels of PA, whereas others may have become more acutely aware of their inability to do so. In line with this, participants reported lower levels of self-efficacy related to PA and experienced more barriers, lower PA levels during leisure time, and a lower HRQoL. Therefore, participants may have felt a stronger need for external support to attain sufficient levels of PA, and thus, had a greater willingness to participate in the trial.

Regarding sociodemographic characteristics, our results are similar to those observed in supervised, noninternet-based exercise trials during and after treatment of prostate, breast, lymphoma, colon, and ovarian cancer [7,11,13]. In line with these studies, participants in our trial were more highly educated than nonparticipants. The relatively high educational level of our total sample may also reflect the fact that the majority of the recruited participants came from the Netherlands Cancer Institute, a specialized oncological treatment center that tends to attract more highly educated patients [28].

A notable finding at baseline was that more than one-third of trial participants reported being in the *maintenance stage* of PA. This was an unexpected finding, as being in this stage (as defined by a short telephone interview) was one of our exclusion criteria. The high number of patients who reported being in the *maintenance stage* could reflect socially desirable responses or overestimation of PA levels, as assessed by the questionnaire. Additional research with objectively measured PA and comprehensive interviews beforehand could be used to investigate whether these biases that apply to the questionnaire or the telephone interview could explain the contradictory results that we observed.

This study has some limitations. First, it is important to place the inclusion rate in the context. First, we invited patients via their treating physicians based on medical record information. Therefore, we were unable to screen survivors on PA levels before sending the invitation. This, in turn, led to approaching many survivors who, in fact, were not eligible for participation because they had sufficient PA levels. This makes it difficult to compare our inclusion rate with that of other semisupervised exercise oncology trials that reported uptake rates of approximately 40% [8,9,11,29]. Second, our findings may, to a certain extent, be subject to recall or social desirability bias. This could have affected the patient-reported outcomes; in particular, some of the nonparticipants may have overreported their levels of PA to justify not participating. Third, selective nonresponses could have occurred where those who were least active also tended not to respond to the nonparticipant questionnaire.

Further research is required that includes survivors with lower educational levels [30]. This group of survivors is expected to be less physically active and thus might benefit more from supportive PA interventions. In addition, the majority of our trial sample was selected from an urbanized region in the Netherlands. A broader multicenter trial could provide results that are more generalizable to breast and prostate cancer survivors living in nonurban areas. Our findings point to a subgroup of patients with an apparent need for support that was self-selected for participation in the trial. Providing appropriate educational materials, timing the offer of interventions to meet the needs of survivors, and having a range of PA interventions (internet-based and supervised) are likely to increase the interest of cancer survivors in such interventions. This holds not only for recruiting survivors into PA intervention studies but also for maximizing the likelihood that they will take up the offer to engage in PA programs offered as a routine element of clinical practice. Finally, efforts should be made to encourage clinicians to follow the recommendations of the American College of Sports Medicine's *Exercise Is Medicine* initiative to assess, advise, and refer patients to exercise or rehabilitation programs [10].

In summary, participants of the PABLO trial showed lower levels of PA before treatment, lower stages of behavioral change, greater symptom burden (most notably fatigue), and a lower level of HRQoL than nonparticipants. These differences between participants and nonparticipants are not reflected in the findings of semisupervised exercise trials that take place during or shortly after treatment. This suggests that the PABLO trial was successful in recruiting cancer survivors who may benefit the most from internet-based supportive PA interventions.

## Abbreviations

HRQoL: health-Related Quality of Life
IPAQ: International Physical Activity Questionnaire
IPAS: Internet-based Physical Activity Support
MFI: Multidimensional Fatigue Inventory
PA: Physical Activity
POMS: Profile of Mood States
RCT: Randomized Controlled Trial
SF-36: Medical Outcomes Study Short Form-36
